# Association between the Alpha-1-acid glycoprotein concentrations and depression in US adult women: a cross-sectional study from NHANES 2021–2023

**DOI:** 10.3389/fpsyt.2025.1555321

**Published:** 2025-06-03

**Authors:** Wanchen Zhao, Hui Zhang, Zhen Li, Cong Ma, Xiaopeng Huo, Na Guo

**Affiliations:** ^1^ Department of Nursing, Peking Union Medical College Hospital, Chinese Academy of Medical Sciences, Beijing, China; ^2^ Department of Clinical Nutrition, Peking Union Medical College Hospital, Chinese Academy of Medical Sciences, Beijing, China

**Keywords:** NHANES, Alpha-1-acid glycoprotein, inflammatory biomarker, depression, cross-sectional study, women

## Abstract

**Background:**

Recent evidence suggests that there is a close correlation of inflammation with depression. Therefore, our study aims to explore the association of Alpha-1-acid glycoprotein (AGP), a highly sensitive inflammatory biomarker, with depression in US adult women.

**Methods:**

Data from the National Health and Nutrition Examination Survey (NHANES) from 2021-2023 were selected for this study. Both AGP concentrations and depression levels were assessed using standardized survey instruments. Multivariate logistic and linear regression, and restricted cubic splines models (RCS) were performed to evaluate the relationship of AGP concentrations with depression. Stratified analyses and multiplicative interaction testing were conducted to evaluate the robustness of the observed association across relevant subgroups.

**Results:**

AGP concentrations demonstrated a positive linear association with depression among US adult women. Each standardized unit increment in ln-transformed AGP concentrations was associated with significantly higher odds of depression (OR = 2.04, 95% CI: 1.17-3.57) and a 1.47-point increase in PHQ-9 scores (β = 1.47, 95% CI: 0.37-2.56) in the fully adjusted model. Furthermore, compared with participants in the lowest AGP concentration quartile, those in the highest quartile exhibited 72% greater odds of depression (OR = 1.72, 95%CI: 1.03-2.87), and 1.32-point higher PHQ-9 scores (*β* = 1.32, 95%CI: 0.31-2.34) in the fully adjusted model. This positive association remained consistent across several subgroups and our sensitivity analysis. In addition, compared to C-reactive protein (CRP), AGP had a stronger predictive effect on depression using the ROC curve.

**Conclusions:**

AGP exhibited a positive linear association with depression in US adult women. This positive association remained consistent across several subgroups. Furthermore, AGP had a stronger predictive effect on depression compared to CRP.

## 1 Introduction

Depression is a debilitating psychiatric condition characterized by persistent depressed mood and marked anhedonia, accompanied by cognitive impairments, psychomotor disturbances, and autonomic dysregulation, collectively contributing to significant somatic dysfunction (1). With the rapid pace of modern life and the increasing pressure of life, more and more people around the world suffer from depression. According to the World Health Organization, nearly 280 million people are diagnosed with depression worldwide, which accounts for approximately 3.8% of the global population (2), of which 10.7% are adults (3, 4). More than 700000 people all around the world annually experience depression-associated suicide (5, 6). Depression brings a substantial public health burden impacting individual well-being, familial functioning, and societal healthcare systems. However, the underlying pathophysiological mechanisms of depression have not been fully explored, but accumulating evidence suggests that inflammatory pathways and related inflammation factors may contribute to the pathogenesis and progression of depression (7, 8).

AGP is a systemic inflammatory biomarker and a non-specific acute-phase reactant, produced primarily by hepatocytes, type II pneumocytes, vascular endothelial cells, and tissue-specific parenchymal cells. After its production, it enters the blood circulation system into plasma, cerebrospinal fluid, gastrointestinal fluid, and urine (9, 10). When the body has inflammation, due to the stimulation of prostaglandin E2 and cytokines, AGP will be synthesized and secreted in large quantities, which has become an effective inflammation biomarker (11).

Emerging evidence demonstrates a significant association between systemic inflammatory markers and clinically diagnosed depression. However, the majority of existing epidemiological studies have primarily examined relationships between interleukin-1b (IL-1b), interleukin-2 (IL-2), interleukin-6 (IL-6), tumor necrosis factor-alpha (TNF-a), and other inflammatory factors and depression (12–16). AGP, as an essential inflammatory marker, can effectively respond to the early inflammation stage in humans. However, there are limited studies that have explored the association of AGP concentrations with depression. The only studies are relatively old and the conclusions have not been unified. For example, Healy D (17) suggested that AGP concentrations were positively correlated with depression risk, but K. Schmid (18) suggested that there was no relationship between them. In addition, recent studies in adolescents by Osuna E (19) and Zarate-Ortiz AG (20) also suggested that iron deficiency could lead to depression. Moreover, inflammatory markers such as C-reactive protein (CRP) and AGP are relatively sensitive indicators to evaluate individual iron status. All these indicate that there may be an association between AGP concentrations and depression, but what kind of association exists and whether this association persists in the adult population still needs to be verified by the study.

Given the existing evidence, a rigorous examination of the association between AGP concentrations and depression using large-scale, nationally representative epidemiological data is warranted. To address this knowledge gap, we conducted a cross-sectional analysis utilizing 2021-2023 NHANES data to investigate AGP-depression associations in the US adult population.

## 2 Methods

### 2.1 Study design and participants

This study conducted a cross-sectional design utilizing data from NHANES, a nationally representative surveillance program employing complex probability sampling methodology. Administered by the Centers for Disease Control and Prevention’s National Center for Health Statistics (CDC/NCHS), NHANES provides comprehensive health and nutritional assessments of the non-institutionalized US population. Participants completed self-administered standardized questionnaires during home visits, followed by comprehensive physical evaluations and clinical laboratory assessments conducted at specially equipped mobile examination centers. All the health-related data for each participant are obtained over the same period and are comparable. The study protocol (#2021-05) received ethical clearance from the Institutional Review Board of the NCHS, with all participants providing documented informed consent before data collection. All the NHANES data are publicly available at https://www.cdc.gov/nchs/nhanes/. Demographic data from the database for 2021-2023 were used in this study.

**Figure 1:**
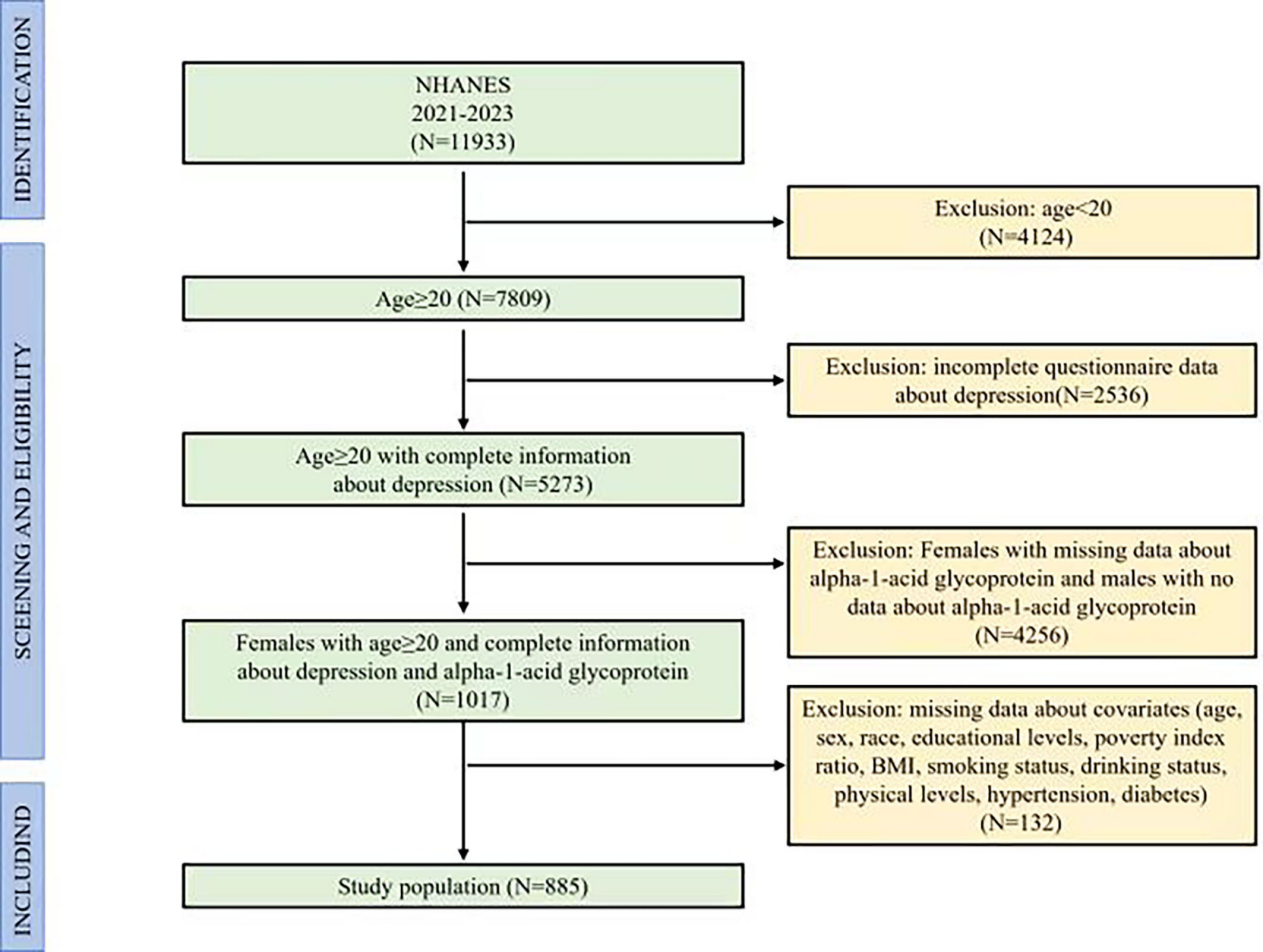
Flow chart of participants in inclusion and exclusion criteria.

The study initially included 11,933 participants. Since the measurement of AGP data in the NHANES (2021–2023) was only presented in adult females, we designed the criteria of inclusion and exclusion for participants, including excluded participants who were males, participants who were females with missing data about AGP, participants with age < 20, participants with incomplete depression screening data, and participants with incomplete covariate measurements. The final study comprised 885 eligible participants, representing a weighted national population estimate of 6.31 million US adults. **Figure 1** illustrated the complete participant selection flowchart, detailing the inclusion/exclusion criteria application.

### 2.2 Measurement of the AGP

The measurement of AGP concentrations was conducted using the Tina-quant Roche AGP assay, which was based on the principle of immunological agglutination. In summary, the AGP antibodies reacted with the antigen in the sample to form an antigen/antibody complex. Following agglutination, this is measured turbidimetrically.

### 2.3 Definitions of depression

Depression was evaluated using the 9-item Public Health Questionnaire (PHQ-9), a validated screening instrument derived from the Diagnostic and Statistical Manual of Mental Disorders, Fourth Edition (DSM-IV) criteria (21, 22). Its nine items directly align with the nine core symptoms of depression in DSM-IV (23). Each item was scored from 0 (not at all) to 3 (nearly every day) based on the frequency of symptom occurrence over the preceding two weeks. Total scores ranged from 0 to 27, with a cutoff of ≥ 10 classifying participants as having depression.

### 2.4 Covariates

In this study, the covariates include age (20-34 years and 35-49 years), race (non-Hispanic White, non-Hispanic Black, Mexican American, or other races), educational levels (lower than high school, high school, and college or above), poverty index ratio (PIR; < 1, 1-3, > 3), body mass index [BMI; < 18.5 kg/m^2^ (underweight), 18.5-24.9 kg/m^2^ (normal weight), 25-29.9 kg/m^2^ (overweight), ≥ 30 kg/m^2^ (obesity)], drinking status (never, former, current) and smoking status (never, former, current), physical activity (vigorous physical level, middle physical level, and others levels), hypertension (yes and no) and diabetes (yes and no) (12) (24). Hypertension was defined as (1): systolic blood pressure ≥ 130 mmHg (2); diastolic blood pressure ≥ 80 mmHg (3); physician-diagnosed hypertension; or (4) current use of antihypertensive medications. Diabetes mellitus was defined as: (1) fasting blood glucose ≥ 126 mg/dL; (2) HbA1c level ≥ 6.5%; (3) physician-diagnosed diabetes; or (4) current use of glucose-lowering medications.

### 2.5 Statistical analysis

Participant baseline characteristics were analyzed using appropriate statistical methods based on variable types: continuous variables were compared using t-tests or the Mann-Whitney U test, while categorical variables were assessed with χ² tests as indicated. To present the distribution condition of AGP concentrations and the potential relationship of AGP concentrations with depression, the dot plots of AGP concentrations in depression and non-depression groups and two-dimensional scattered plots of the correlation between AGP concentrations and PHQ-9 scores were performed. To reduce the skew of the AGP concentrations in subsequent statistical analysis, the AGP concentrations were ln-transformed. The AGP concentrations were regarded as continuous and category variables. When it was category variables, it was divided into the fourth quartile (the lowest quartile, the second and third quartiles, and the highest quartile). The associations between natural logarithm-transformed AGP concentrations and both depression status (via weighted multivariable-adjusted logistic regression) and continuous symptom severity scores (via weighted linear regression) were examined, with restricted cubic splines (RCS) modeling employed to evaluate potential nonlinear dose-response relationships. To evaluate the robustness of the observed association between AGP concentrations and depression, stratified analyses, interaction tests, and sensitivity analyses were performed. The Benjamini-Hochberg (BH) method was used to adjust the multiple comparison corrections to cause potential the risk of false positives to increase. To comprehensively assess the predictive capacity of AGP for depression, CRP was incorporated as a comparator inflammatory marker. Receiver operating characteristic (ROC) curve analysis was then performed to evaluate and compare the diagnostic accuracy of both biomarkers. All statistical analyses were conducted using R statistical software (v4.3.1; R Foundation for Statistical Computing), with statistical significance determined *a priori* as two-tailed *P* < 0.05. This study employed a cross-sectional design utilizing 2021-2023 NHANES data to examine associations between AGP concentrations and clinically assessed depression, which may not establish causal relationships due to this study design.

## 3 Result

### 3.1 Baseline demographic characteristics of the participants

**Figure 2:**
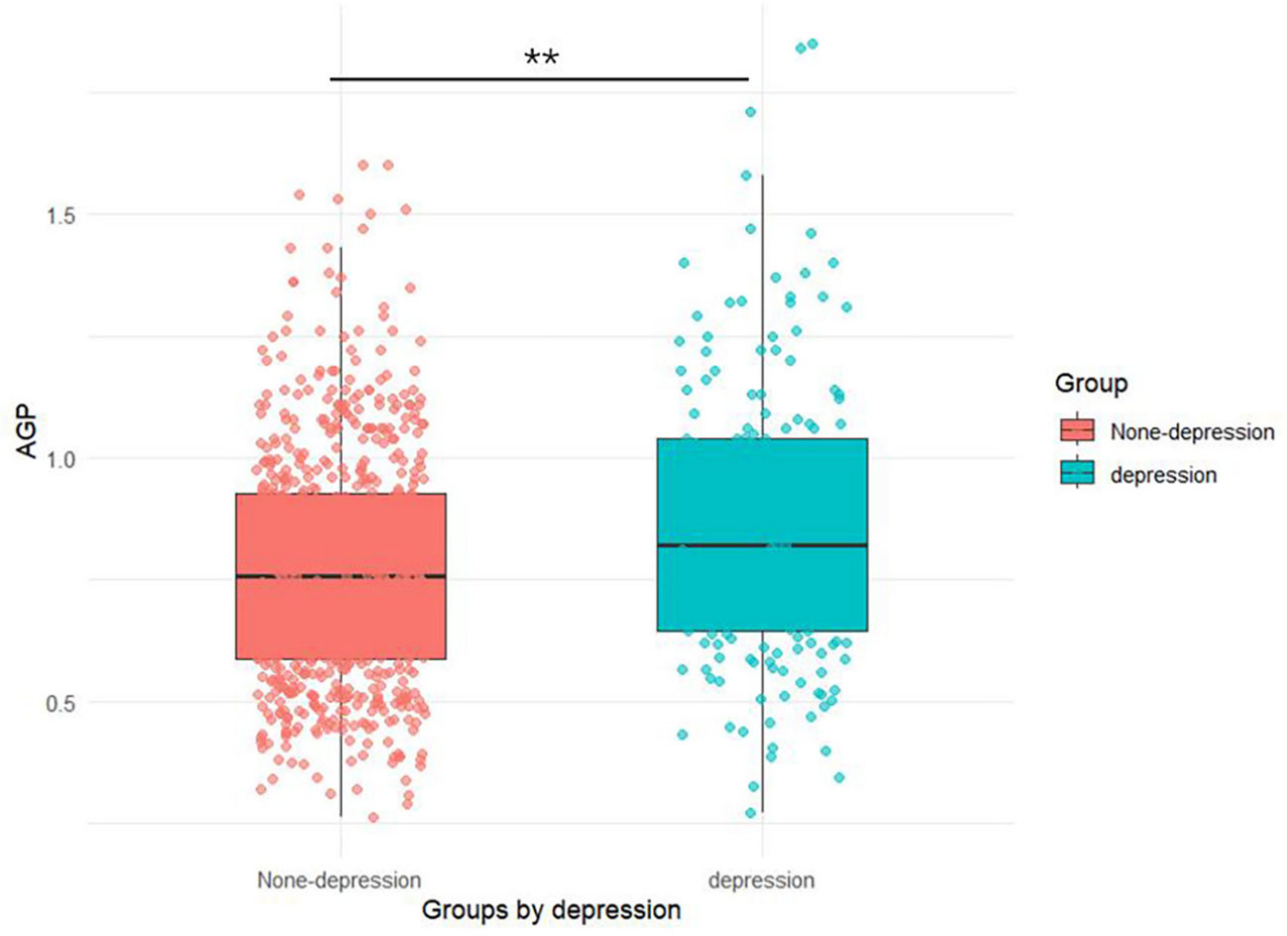
Dot plots of AGP concentrations in depression and non-depression groups.

As presented in **Table 1**, our study included 885 women aged ≥ 20 years, comprising 183 (20.7%) with depression and 702 (79.3%) without depression. The AGP concentrations (0.86 ± 0.28) in depression groups were higher than those (0.77 ± 0.24) in none-depression groups in **Figure 2**. Mann-Whitney U tests, t-tests and chi-square tests revealed significant intergroup differences in multiple variables including socioeconomic status (PIR), anthropometric measures (BMI), health behaviors (tobacco use and physical activity), cardiometabolic comorbidities (hypertension and diabetes), inflammatory markers (AGP concentrations), and depressive symptom severity (PHQ-9 scores) (all *P* < 0.05).

**Table 1:** Baseline demographic characteristics of the participants.

Characteristic	Total (n = 885), n (%) or mean (se)	Depression (n = 183), n (%) or mean (se)	None-depression (n = 702), n (%) or mean (se)	*P*-value
Age				0.311
20-34 years	412 (46.55)	92 (50.27)	320(46.92)	
35-49 years	473 (53.45)	91 (49.73)	382 (53.08)	
Race				0.622
Non-Hispanic White	478 (54.02)	107 (58.46)	371 (54.39)	
Non-Hispanic Black	113 (12.76)	20 (10.94)	93 (13.63)	
Mexican American	74 (8.36)	14 (7.65)	60 (8.79)	
Other	220 (24.86)	42 (22.95)	178 (26.09)	
Educational levels				0.121
Less than High-school	66 (7.45)	19 (10.38)	47 (6.69)	
High school	133 (15.04)	32 (17.48)	101 (14.38)	
College or above	686 (77.51)	132 (72.14)	554 (78.93)	
PIR				< 0.001
PIR < 1	156 (17.62)	47 (25.68)	109 (15.54)	
1 ≤ PIR < 3	350 (39.54)	91 (49.73)	259 (36.89)	
PIR > 3	379 (42.84)	45 (24.59)	334 (47.57)	
BMI				< 0.001
Underweight	14 (1.58)	6 (3.27)	8 (1.13)	
Normal weight	259 (29.26)	39 (21.32)	220 (31.33)	
Overweight	255 (28.82)	43 (23.49)	212 (30.19)	
Obesity	357 (40.34)	95 (51.92)	262 (37.32)	
Drinking status				0.411
Never	69 (7.79)	18 (9.83)	51 (7.26)	
Former	70 (7.92)	17 (9.28)	53 (7.56)	
Current	746 (84.29)	148 (80.89)	598 (85.18)	
Smoking status				< 0.001
Never	627 (70.84)	110 (60.10)	517 (73.64)	
Former	151 (17.06)	37 (20.23)	114 (16.23)	
Current	107 (12.10)	36 (19.67)	71 (10.11)	
Physical levels				0.005
Vigorous	476 (53.78)	79 (43.16)	397 (56.56)	
Middle	277 (31.29)	68 (37.17)	209 (29.77)	
Other	132 (14.93)	36 (19.67)	96 (13.67)	
Hypertension				0.004
Yes	270 (30.50)	73 (39.89)	197 (28.07)	
No	615 (69.50)	110 (60.11)	505 (71.93)	
Diabetes				0.008
Yes	70 (7.90)	24 (13.11)	46 (6.55)	
No	815 (92.10)	159 (86.89)	656 (93.44)	
AGP (g/L)				< 0.001
	0.79 ± 0.25	0.86 ± 0.28	0.77 ± 0.24	
PHQ-9 score				< 0.001
	5.72 ± 5.38	14.2 ± 4.30	3.50 ± 2.79	

Categorical variables were presented as percentages, and the chi-square test was used to detect the statistical difference between them. Continuous categorical variables were presented as mean ± se, and the t-test was used to detect the statistical difference in the PHQ-9 scores, and the Mann-Whitney U test was used to detect the statistical difference in the AGP concentrations.

### 3.2 Association between AGP concentrations and depression

**Figure 3:**
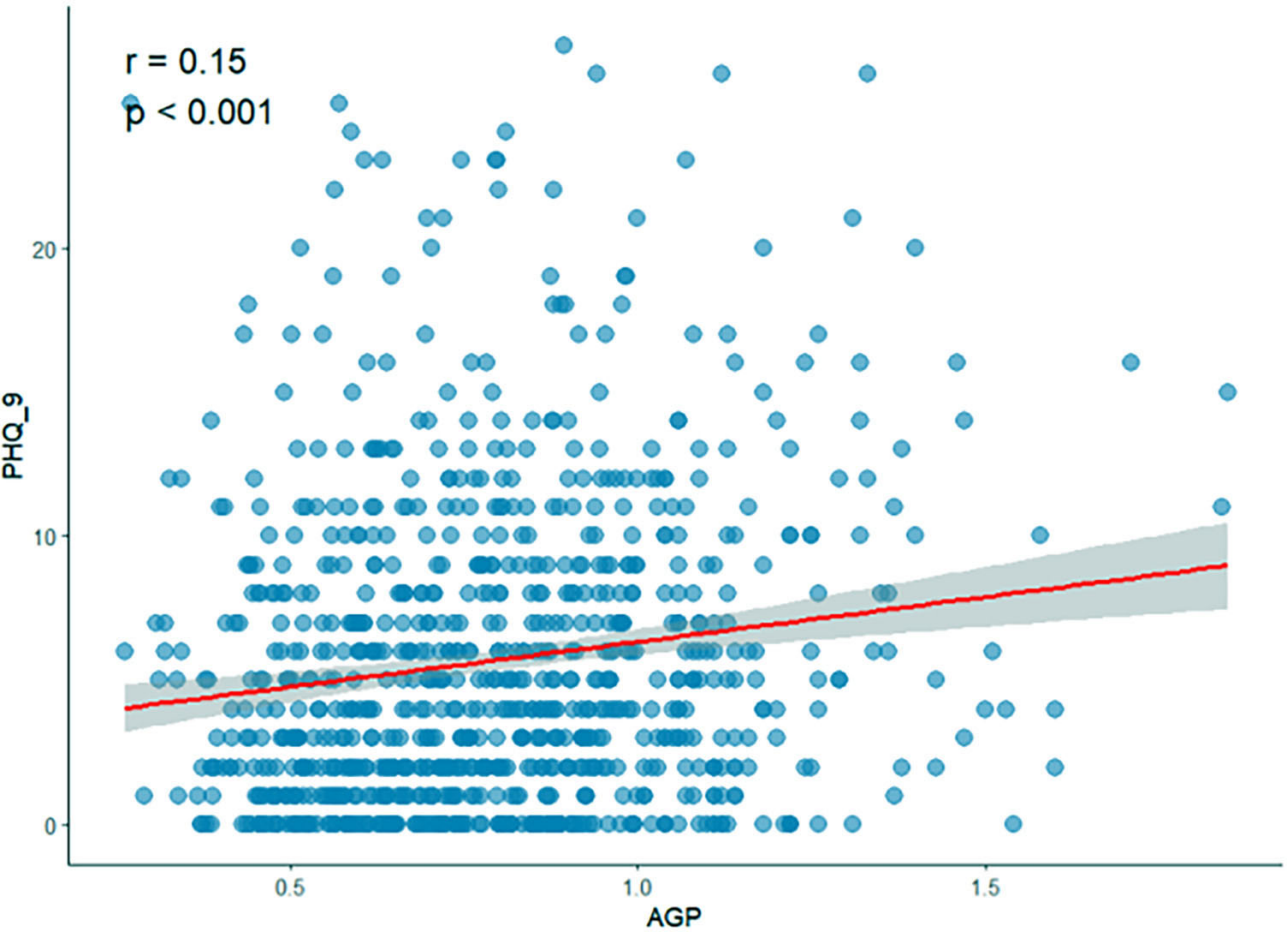
Two-dimensional scattered plots of correlation between AGP concentrations and PHQ-9 score.

**Figure 4:**
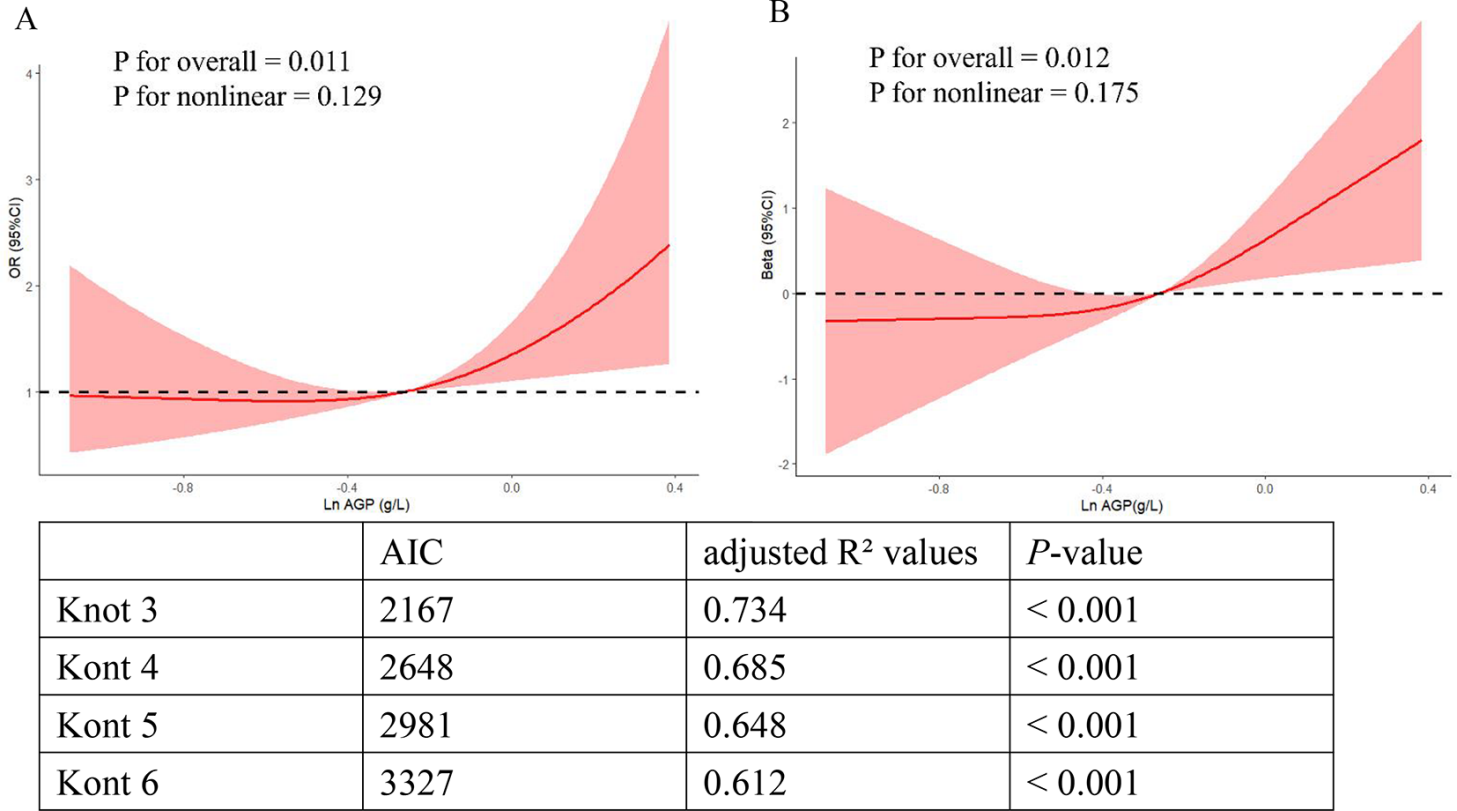
The linear relationship of ln-transformed AGP with depression and the PHQ-9 scores in RCS models. **(A)** The linear relationship of ln-transformed AGP with the depression risk. **(B)** The linear relationship of ln-transformed AGP with the PHQ-9 scores.

The two-dimensional scattered plots between AGP concentrations and PHQ-9 scores were performed to initially reveal the potential relationship, this result showed that AGP concentrations may have a positive relationship with PHQ-9 scores (r = 0.15, *P* < 0.001) (**Figure 3**). Therefore, to further examine the association, the weighed multivariates logistical regression and linear models and RCS models were used. **Table 2** demonstrated the significant association of AGP concentrations with depression. Each ln-unit increase in AGP concentrations was associated with significantly higher odds of depression across progressively adjusted models: Model 1 (OR = 2.87, 95%CI: 1.69-4.86), Model 2 (OR = 2.35, 95%CI: 1.36-4.06), and Model 3 (OR = 2.04, 95%CI: 1.17-3.57). Consistently, participants in the uppermost quartile of AGP concentrations demonstrated a significantly elevated odds ratio for depression relative to those in the lowest quartile: Model 1 (OR = 2.36, 95%CI: 1.46-3.82), Model 2 (OR = 1.95, 95%CI: 1.18-3.23), and Model 3 (OR = 1.72, 95%CI = 1.03-2.87). Notably, consistent positive associations were observed between AGP concentrations and PHQ-9 scores. To further reveal whether there was a significant linear relationship between AGP and depression. A trend analysis was used, and our results showed an increased trend of the odds ratio of depression with AGP concentrations in three models (all *P* for trend < 0.05). The RCS models with three knots [adjusted R2 value was the highest (0.734) and AIC value was lowest (2167), which suggested that the model in this condition had the best performance] were performed to further explore the potential non-linear relationship between AGP concentrations and depression and PHQ-9 scores. Our results showed a linear relationship between ln-transform AGP and depression (*P* for overall = 0.011, *P* for nonlinear = 0.129) and the PHQ-9 scores (*P* for overall = 0.012, *P* for nonlinear = 0.175), respectively, which was consistent with our linear trend analysis (**Figure 4**).

**Table 2:** Association between AGP concentrations and depression and PHQ-9 scores.

	OR (95% CI)	*P*-value	*β* (95% CI)	*P*-value
Crude model (Model 1)				
Continuous	2.87 (1.69, 4.86)	< 0.001	2.23 (1.15, 3.31)	< 0.001
Q1	*Ref* (1.00)		*Ref* (0.00)	
Q2	1.45 (0.87, 2.41)	0.144	0.72 (-0.26, 1.72)	0.151
Q3	1.59 (0.96, 2.62)	0.067	1.25 (0.26, 2.25)	0.014
Q4	2.36 (1.46, 3.82)	< 0.001	2.05 (1.05, 3.05)	< 0.001
*P* for trend	< 0.001		< 0.001	
partially adjusted model (Model 2)				
Continuous	2.35 (1.36, 4.06)	0.002	1.84 (0.76,2.92)	< 0.001
Q1	*Ref* (1.00)		*Ref* (0.00)	
Q2	1.35 (0.81, 2.27)	0.247	0.61 (-0.35, 1.58)	0.212
Q3	1.49 (0.89, 2.49)	0.123	1.14 (0.16, 2.12)	0.022
Q4	1.95 (1.18, 3.23)	0.008	1.65 (0.66, 2.65)	0.001
*P* for trend	0.008		< 0.001	
Fully adjusted model (Model 3)				
Continuous	2.04 (1.17, 3.57)	0.011	1.47 (0.37, 2.56)	0.008
Q1	*Ref* (1.00)		*Ref* (0.00)	
Q2	1.27 (0.75, 2.13)	0.368	0.47 (-0.49,1.44)	0.339
Q3	1.36 (0.81, 2.28)	0.242	0.91 (-0.06, 1.90)	0.066
Q4	1.72 (1.03, 2.87)	0.032	1.32 (0.31, 2.34)	0.010
*P* for trend	0.036		0.006	

Reference. Model I, no covariates were adjusted. Model II, age, race, education levels, or PIR were adjusted. Model III, all covariates were adjusted.

### 3.3 Stratified analyses and sensitivity analyses

To evaluate the stability of the observed relationship between depression and AGP concentrations, we performed comprehensive stratified analyses and multivariable sensitivity testing across clinically relevant subgroups. To control the multiple comparisons to increase the risk of false positives, we used the method of Benjamini-Hochberg (BH) to adjust all *P*-values in stratified analyses (**Supplementary Table S1**). Our results of stratified analyses showed consistent positive associations of AGP concentrations with depression and PHQ-9 values across specified demographic and clinical subgroups, such as younger adults (20-34 years), non-Hispanic White participants, college-educated participants, middle-income groups (PIR 1-3), current alcohol consumers, non-smokers, physically active individuals (vigorous level), Non-diabetic participants (*P* < 0.05, adjusted *P* < 0.05). Additionally, stratified analyses revealed no significant effect modification by AGP concentrations on depression (all interaction *P*-values > 0.05) (**Table 3**). To address potential bias from missing covariates in the association of AGP concentrations with depression, we employed multiple imputation methods for missing data handling. The results demonstrated robust stability of the positive AGP-depression association across all analytical models (**Supplementary Table S2**).

**Table 3:** The associations between ln transform AGP concentrations, depression, and the PHQ-9 scores in subgroups.

Subgroups	OR (95% CI)	*P*-value	*P* for interaction	*β* (95% CI)	*P*-value	*P* for interaction
Age			0.695			0.250
20-34 years	2.34 (1.06, 5.14)	0.034		1.99 (0.49, 3.49)	0.009	
35-49 years	1.91 (0.82, 4.41)	0.128		0.85 (-0.77, 2.49)	0.303	
Race			0.423			0.271
Non-Hispanic White	2.66 (1.25, 5.67)	0.011		2.06 (0.56, 3.57)	0.007	
Non-Hispanic Black	0.51 (0.09, 2.92)	0.454		-1.45(-4.09, 1.18)	0.282	
Mexican American	2.22 (0.42, 9.00)	0.183		3.62 (-1.63, 8.87)	0.181	
Other	1.39 (0.44, 4.31)	0.566		0.61 (-1.61, 2.85)	0.589	
Educational levels			0.962			0.642
Less than High-school	1.12 (0.11, 10.65)	0.924		-1.88 (-7.47, 3.71)	0.512	
High school	2.78 (0.63, 12.23)	0.174		2.11 (-0.93, 5.16)	0.176	
College or above	2.11 (1.10, 4.03)	0.023		1.47 (0.26, 2.68)	0.017	
Poverty index ratio			0.324			0.111
PIR < 1	0.41 (0.12, 1.39)	0.152		-3.74 (-7.09, -0.38)	0.031	
1 ≤ PIR < 3	5.71 (2.43, 13.42)	< 0.001		3.69 (1.85, 5.52)	< 0.001	
PIR > 3	1.50 (0.51, 4.41)	0.457		0.94 (-0.42, 2.31)	0.178	
BMI			0.861			0.644
Underweight	1.49 (0.25, 7.60)	0.291		0.94 (0.25, 3.76)	0.025	
Normal weight	2.10 (0.66, 6.58)	0.203		0.56 (-1.31, 2.45)	0.554	
Overweight	0.63 (0.18, 2.25)	0.484		0.87 (-1.46, 3.22)	0.463	
Obesity	2.32 (0.78, 6.83)	0.126		1.09 (-1.29, 3.47)	0.371	
Drinking status			0.643			0.681
Never	1.07 (0.14, 8.09)	0.950		0.46 (-3.92, 4.85)	0.836	
Former	0.61 (0.08, 4.58)	0.636		-0.27 (-4.67, 4.12)	0.901	
Current	2.94 (1.55, 5.57)	< 0.001		2.01 (0.83, 3.18)	< 0.001	
Smoking status			0.474			0.193
Never	2.13 (1.07, 4.24)	0.031		1.78 (0.54, 3.03)	0.004	
Former	0.95 (0.26, 3.46)	0.949		-1.34 (-4.19, 1.51)	0.359	
Current	3.48 (0.88, 14.11)	0.067		2.19 (-2.04, 6.44)	0.312	
Physical levels			0.089			0.642
Vigorous	2.89 (1.25, 6.68)	0.012		1.51 (0.12, 2.91)	0.033	
Middle	1.93 (0.75, 4.95)	0.170		1.57 (-0.54, 3.69)	0.146	
Other	1.93 (0.43, 8.66)	0.607		0.44 (-3.21, 4.09)	0.813	
Hypertension			0.939			0.654
Yes	2.26 (0.82, 6.21)	0.112		0.98 (-1.46, 3.42)	0.431	
No	1.95 (0.99, 3.85)	0.052		1.71 (0.49, 2.91)	0.005	
Diabetes			0.856			0.956
Yes	2.42 (0.53, 15.23)	0.153		3.26 (-2.24, 8.77)	0.252	
No	2.08 (1.16, 3.73)	0.013		1.46 (0.33, 2.58)	0.011	

Age, race, educational levels, PIR, BMI, drinking status, smoking status, physical levels, hypertension, and diabetes were adjusted in the Model III.

### 3.4 The prognostic value of AGP concentrations for depression

**Figure 5:**
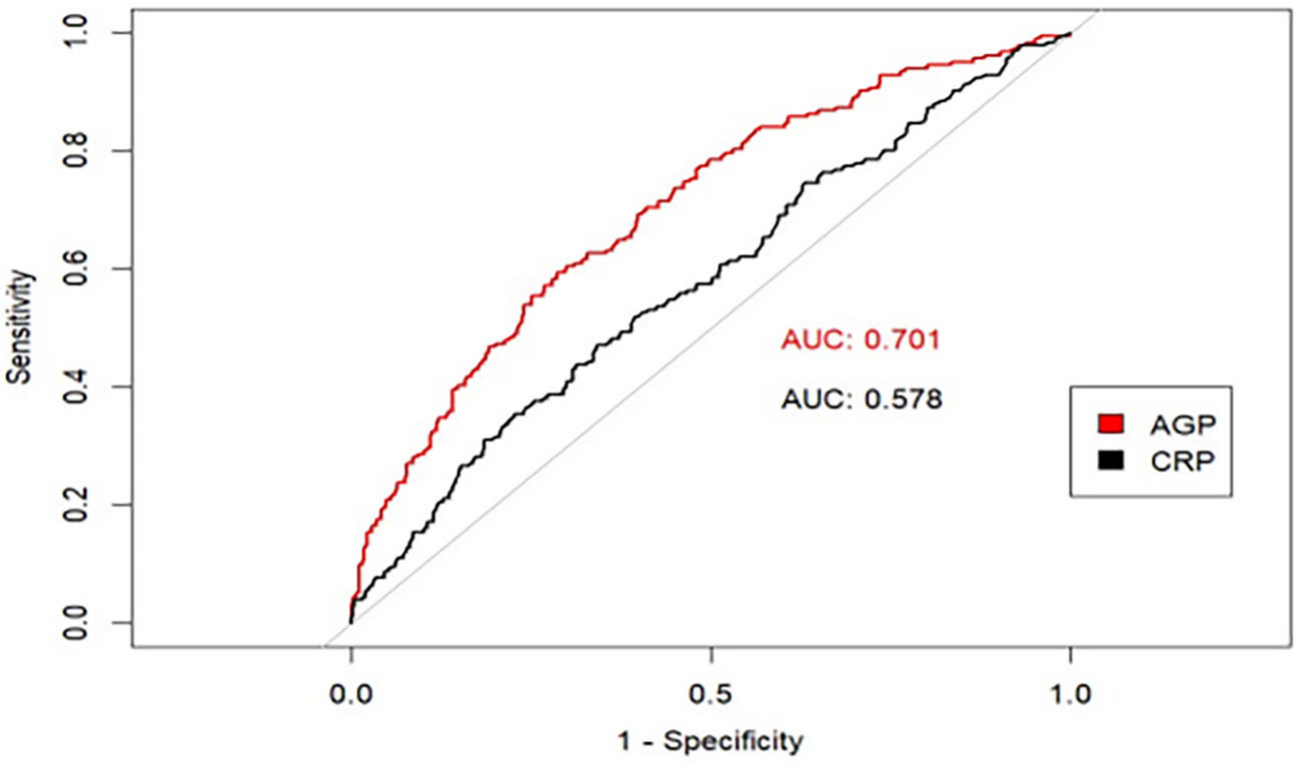
The predictive effect of AGP using ROC curves.

To evaluate the prognostic capacity of AGP concentrations for clinical depression diagnosis, the ROC curves were employed to assess the prognostic capacity of AGP for depression risk prediction. Since the CRP has been demonstrated it had a great predictive effect on depression (25–27). Therefore, in our study, the CRP was used to be an inflammatory biomarker reference. The analytical results demonstrated that AGP concentrations exhibited superior prognostic accuracy for depression compared to CRP, as a conventional risk predictor (AUC: 0.701 vs 0.578, *P* < 0.001) (**Figure 5**).

## 4 Discussion

Some epidemiology and animal studies have shown that there is a close link between inflammation and depression, the levels of proinflammatory cytokines such as interleukin-1b (IL-1b), interleukin-2 (IL-2), interleukin-6 (IL-6), tumor necrosis factor-alpha (TNF-a) and anti-inflammatory cells such as interleukin-4 (IL-4), interleukin-10 (IL-10) are higher in patients with depression (12–16). Inflammatory mediators can affect monoamine transmitters and cause changes in glutamate neurotransmission, glucocorticoid receptor resistance, etc. These molecular mediators constitute key pathophysiological substrates in depression etiology. Furthermore, pro-inflammatory cytokines can disrupt neural circuitry signaling, contributing to neurocognitive dysfunction and core depressive symptomatology, including persistent anhedonia and depressed mood (28–31). In addition, some evidence has supported that some pro-inflammatory factors, such as IL-1, IL-6, and TNF-α, could increase the synthesis of acute phase reactant AGP (32). Most present studies mainly focused on exploring the relationship of IL-1b, IL-2, IL-6, and TNF-a, with depression. However, the relationship of AGP concentrations with depression risk is controversial at present.

This cross-sectional study utilized 2021-2023 NHANES data to examine associations between AGP concentrations and depression. Multivariable-adjusted analyses, including logistic regression (categorical depression outcome), linear regression (continuous PHQ-9 scores), and restricted cubic splines (nonlinear relationships), consistently demonstrated significant positive linear associations. These associations remained robust across key demographic and clinical strata: age groups, racial categories, educational attainment, PIR tertiles, substance use status (tobacco/alcohol), physical activity levels, and diabetes status (all interaction *P*-values > 0.05).

Although the precise pathophysiological mechanism of the association between AGP concentrations and depression has not been elucidated, the underlying mechanism cannot be explained without the inflammatory and neuroprogressive (IN-PRO) hypothesis of depression. In this hypothesis, cell-mediated immune activation and inflammatory pathways and their consequences or accompanying symptoms are implicated as the cause of the neuro-progressive process, which in turn can lead directly to the development of depression (33). AGP gene expression is controlled by a combination of major regulatory mediators IL-1, IL-6, and TNF-α. IL-1, IL-6, and TNF-α, on the other hand, lead to neural progression and depression through their pathways (33). For example, IL-1 induces depression by decreasing the mRNA and protein levels of BDNF and NGF, and decreasing the expression of the neurotrophin TrK receptor in the hippocampus, affecting the survival of neurons (34). IL-6 leads to neural progression by promoting astrocyte apoptosis and contributes to the occurrence of depression (35). TNF-αleads to neural progression through a variety of pathways such as caspase-dependent cascades, enhanced glutamate neurotoxicity, or stimulation of glutamate release from microglia by upregulating glutaminase and blocking glutamate transporter activity, as well as silencing cell survival signals, which further leads to the occurrence of depression (36).

Our results are consistent with the results of BahriniL.et al. (37), which studied the association of AGP concentrations with depression in the Middle Eastern and North African populations. However, our results were different from the results of K. Schmid. et al. (18), which revealed that the relationship between them was not statistically significant, the reason may be that K. Schmid’s research was published earlier and the technology and methods of AGP detection at that time were not advanced, which induced detected AGP concentrations might not represent the real AGP concentrations in the general population. In addition to this, from a methodological perspective, the reason for the difference between our research and K. Schmid’s research may be that the sample size of K. Schmid’s research was too small and the statistical power was insufficient to detect the difference.

Some studies have demonstrated that inflammatory factors such as IL-6 and TNF-α may be difficult to evaluate in chronic inflammatory states owing to their rapid pharmacokinetic characteristics, but AGP demonstrates measurable concentrations across diverse physiological and pathological conditions owing to its distinct intermediate pharmacokinetic profile. Thus, AGP was considered an essential inflammatory marker in acute inflammatory states, and it may be more sensitive in the examination of inflammation levels. Our study showed that in terms of predictive performance, the area under the ROC curve of AGP for depression was 0.70, which was much larger than the area under the ROC curve of other inflammatory markers such as CRP (0.58) and HDL-related inflammation indexes (0.65) (38). This proves that AGP has good predictive performance and clinical applicability and application prospects for depression. In addition, some studies have shown that some patients’ depressive symptoms were also relieved during the treatment with anti-inflammatory factors (39, 40). The results of our study provide some insights into the anti-inflammatory treatment of depression by reducing AGP concentrations and other inflammatory markers. Therefore, in the following studies, we can try to explore whether drugs that reduce AGP concentrations can be used to relieve depressive symptoms.

This study possesses several methodological advantages (1): enhanced population representativeness: utilizing a substantially larger, nationally representative sample compared to prior studies (weighted n = 6.31 million), our findings demonstrate superior generalizability and statistical power (2); standardized biomarker assessment: AGP concentrations are quantified using standardized immunoturbidimetric assays performed in CDC-certified laboratories, ensuring analytical precision superior to conventional methodologies (3). robust confounding control: Multivariable adjustment for demographic, behavioral, and clinical covariates (including age, race/ethnicity, socioeconomic status, cardiometabolic factors, and lifestyle variables) was implemented, with sensitivity analyses confirming result stability.

Our study also had certain limitations. Firstly, since AGP data was presented only in females, the participants included in this study were only females, and there might be a gender bias. Secondly, the cross-sectional study design used in this study had its limitations in that causality could not be determined. It limited the possibility of establishing a causal relationship between AGP concentrations and depression risk. Specifically, through the current study, we cannot determine whether higher AGP concentrations lead to the development of depression or vice versa. Thirdly, the study included unadjusted confounders such as diet, chronic medical conditions, and psychosocial factors, which may have introduced bias and affected the stability of these results in this study. Fourthly, this study relied on NHANES data and lacked an assessment of biological variability in AGP, including individual differences and diurnal fluctuations, which may reduce the generalisability of the conclusions. In addition, longitudinal follow-up of AGP was lacking in this study to determine whether changes in AGP concentrations were associated with depression progression. Consequently, well-designed longitudinal cohort studies incorporating repeated biomarker measurements, complemented by Mendelian randomization analyses leveraging genetic instrumental variables, are warranted to establish causal inference regarding this association. In addition, some animal studies are needed to explore the potential biological mechanisms in this process.

## 5 Conclusion

In summary, this study demonstrates a significant dose-dependent relationship between AGP concentrations and depression in adult women based on the NHANES database. In the future, more longitudinal and animal studies deserve to further explore potential biological mechanisms between them.

## Data Availability

The original contributions presented in the study are included in the article/supplementary material. Further inquiries can be directed to the corresponding author.

## References

[1] Evans-Lacko S Aguilar-Gaxiola S Al-Hamzawi A Alonso J Benjet C Bruffaerts R. Socio-economic variations in the mental health treatment gap for people with anxiety, mood, and substance use disorders: results from the WHO World Mental Health (WMH) surveys. Psychol Med. (2018) 48:1560–71. doi: 10.1017/s0033291717003336 PMC6878971 29173244

[2] Ferrari AJ Santomauro DF Herrera AMM Shadid J Ashbaugh C Erskine HE. Global regional and national burden of 12 mental disorders in 204 countries and territories, 1990-2019: a systematic analysis for the Global Burden of Disease Study 2019. Lancet Psychiatry. (2022) 9 (2):137–50. doi: 10.1016/s2215-0366(21)00395-3 PMC8776563 35026139

[3] Vos T Lim SS Abbafati C Abbas KM Abbasi M Abbasifard M. Global burden of 369 diseases and injuries in 204 countries and territories, 1990-2019: a systematic analysis for the Global Burden of Disease Study 2019. Lancet. (2020) 396:1204–22. doi: 10.1016/s0140-6736(20)30925-9 PMC7567026 33069326

[4] World Health Organization. Depression E. coli (2023). Available online at: https://www.who.int/zh/news-room/fact-sheets/detail/depression (Accessed November 30, 2024).

[5] Institute of Health Metrics and Evaluation. Global health dataexchange(GHDx). E. coli (2023). Available online at: http://ghdx.healthdata.org/gbd-results-tool?params=gbd-api-2019-permalink/d780dffbe8a381b25e1416884959e88b (Accessed November 30, 2024).

[6] Smith K. Mental health: a world of depression. Nature. (2014) 515:181. doi: 10.1038/515180a 25391942

[7] Milaneschi Y Kappelmann N Ye Z Lamers F Moser S Jones PB. Association of inflammation with depression and anxiety: evidence for symptom-specificity and potential causality from UK Biobank and NESDA cohorts. Mol Psychiatry. (2021) 26:7393–402. doi: 10.1038/s41380-021-01188-w PMC8873022 34135474

[8] Zunszain PA Hepgul N Pariante CM. Inflammation and depression. Curr Top Behav Neurosci. (2013) 14:135–51. doi: 10.1007/7854_2012_211 22553073

[9] Sierra T González MC Moreno B Crevillen AG Escarpa A. Total α(1)-acid glycoprotein determination in serum samples using disposable screen-printed electrodes and osmium (VI) as electrochemical tag. Talanta. (2018) 180:206–10. doi: 10.1016/j.talanta.2017.12.018 29332802

[10] Yoo J Yoon T Park YB Ahn SS Lee SW. The Clinical Utility of Serum Alpha-1-Acid Glycoprotein in Reflecting the Cross-Sectional Activity of Antineutrophil Cytoplasmic Antibody-Associated Vasculitis: A Single-Centre Retrospective Study. Medicina (Kaunas). (2024) 60 (8):1212. doi: 10.3390/medicina60081212 39202493 PMC11356503

[11] Fournier T Bouach N Delafosse C Crestani B Aubier M. Inducible expression and regulation of the alpha 1-acid glycoprotein gene by alveolar macrophages: prostaglandin E2 and cyclic AMP act as new positive stimuli. J Immunol. (1999) 163:2883–90. doi: 10.4049/jimmunol.163.5.2883 10453035

[12] Mazza MG De Lorenzo R Conte C Poletti S Vai B Bollettini I. Anxiety and depression in COVID-19 survivors: Role of inflammatory and clinical predictors. Brain Behav Immun. (2020) 89:594–600. doi: 10.1016/j.bbi.2020.07.037 32738287 PMC7390748

[13] Köhler CA Freitas TH Maes M De Andrade NQ Liu CS Fernandes BS. Peripheral cytokine and chemokine alterations in depression: a meta-analysis of 82 studies. Acta Psychiatr Scand. (2017) 135:373–87. doi: 10.1111/acps.12698 28122130

[14] Nusslock R Brody GH Armstrong CC Carroll AL Sweet LH Yu T. Higher peripheral inflammatory signaling associated with lower resting-state functional brain connectivity in emotion regulation and central executive networks. Biol Psychiatry. (2019) 86:153–62. doi: 10.1016/j.biopsych.2019.03.968 PMC7430716 31054766

[15] Duivis HE Vogelzangs N Kupper N De Jonge P Penninx BW. Differential association of somatic and cognitive symptoms of depression and anxiety with inflammation: findings from the Netherlands Study of Depression and Anxiety (NESDA). Psychoneuroendocrinology. (2013) 38:1573–85. doi: 10.1016/j.psyneuen.2013.01.002 23399050

[16] Peters AT Ren X Bessette KL George N Kling LR Thies B. Inflammation, depressive symptoms, and emotion perception in adolescence. J Affect Disord. (2021) 295:717–23. doi: 10.1016/j.jad.2021.08.126 PMC8551069 34517245

[17] Healy D Calvin J Whitehouse AM White W Wilton-Cox H Theodorou AE. Alpha-1-acid glycoprotein in major depressive and eating disorders. J Affect Disord. (1991) 22:13–20. doi: 10.1016/0165-0327(91)90078-7 1652602

[18] Putnam FW. The plasma proteins: structure, function and genetic control. New York: Academic Press (1975) p. 184–228.

[19] Osuna E Baumgartner J Wunderlin O Emery S Albermann M Baumgartner N. Omega-3 Study Team. Iron status in Swiss adolescents with paediatric major depressive disorder and healthy controls: a matched case-control study. Eur J Nutr. (2024) 63:951–63. doi: 10.1007/s00394-023-03313-7 PMC10948461 38265750

[20] Zarate-Ortiz AG Verhoef H Melse-Boonstra A Woods BJ Lee-Bazaldúa EE Feskens EJ. Depressive symptoms among Mexican adolescent girls in relation to iron status, anaemia, body weight and pubertal status: results from a latent class analysis. Public Health Nutr. (2023) 26:408–15. doi: 10.1017/S1368980022001203 PMC13076068 35583048

[21] Ji Y Wang J. Association between blood cadmium and depression varies by age and smoking status in US adult women: a cross-sectional study from NHANES 2005-2016. Environ Health Prev Med. (2024) 29:32. doi: 10.1265/ehpm.24-00050 38910137 PMC11211073

[22] Hu PW Zhang XL Yan XT Qi C Jiang GJ. Association between depression and endometriosis using data from NHANES 2005-2006. Sci Rep. (2023) 13:18708. doi: 10.1038/s41598-023-46005-2 37907559 PMC10618216

[23] Muñoz-Navarro R Cano-Vindel A Medrano LA Schmitz F Ruiz-Rodríguez P Abellán-Maeso C. Utility of the PHQ-9 to identify major depressive disorder in adult patients in Spanish primary care centres. BMC Psychiatry. (2017) 17:291. doi: 10.1186/s12888-017-1450-8 28793892 PMC5550940

[24] Xue Y Xu J Li M Gao Y. Potential screening indicators for early diagnosis of NAFLD/MAFLD and liver fibrosis: Triglyceride glucose index-related parameters. Front Endocrinol (Lausanne). (2022) 13:951689. doi: 10.3389/fendo.2022.951689 36120429 PMC9478620

[25] Tavares VDO Schuch FB de Sousa GM Hallgren M Teychenne M de Almeida RN. Does multimodal exercise reduce C-reactive protein levels in major depressive disorder? Preliminary results from a randomized controlled trial. J Psychiatr Res. (2025) 183:252–9. doi: 10.1016/j.jpsychires.2025.02.023 40010075

[26] Zhang T Ji C Zhu J Wang X Shen C Liang F. Comparison of clinical features and inflammatory factors between patients with bipolar depression and unipolar depression. BMC Psychiatry. (2025) 25:108. doi: 10.1186/s12888-025-06516-w 39930379 PMC11812187

[27] Stephenson AR Ka-Yi Chat I Bisgay AT Coe CL Abramson LY Alloy LB. Higher inflammatory proteins predict future depressive symptom severity among adolescents with lower emotional clarity. Brain Behav Immun. (2024) 122:388–98. doi: 10.1016/j.bbi.2024.08.035 PMC11418926 39163913

[28] Zaki NFW Spence DW Bahammam AS Pandi-Perumal SR Cardinali DP Brown GM. Chronobiological theories of mood disorder. Eur Arch Psychiatry Clin Neurosci. (2018) 268:107–18. doi: 10.1007/s00406-017-0835-5 28894915

[29] Moorman AJ Mozaffarian D Wilkinson CW Lawler RL Mcdonald GB Crane BA. In patients with heart failure elevated soluble TNF-receptor 1 is associated with higher risk of depression. J Card Fail. (2007) 13:738–43. doi: 10.1016/j.cardfail.2007.06.301 17996822

[30] Suarez EC Krishnan RR Lewis JG. The relation of severity of depressive symptoms to monocyte-associated proinflammatory cytokines and chemokines in apparently healthy men. Psychosom Med. (2003) 65:362–8. doi: 10.1097/01.psy.0000035719.79068.2b 12764208

[31] Walker AK Kavelaars A Heijnen CJ Dantzer R. Neuroinflammation and comorbidity of pain and depression. Pharmacol Rev. (2014) 66:80–101. doi: 10.1124/pr.113.008144 24335193 PMC3880465

[32] Vasileiadou K Pantazidis G Papadopoulou K Ligoudistianou C Kourelis A Petrakis S. alpha1-Acid glycoprotein production in rat dorsal air pouch in response to inflammatory stimuli, dexamethasone and honey bee venom. Exp Mol Pathol. (2010) 89 (1):63–71. doi: 10.1016/j.yexmp.2010.03.008 20363221 PMC2900460

[33] Leonard B Maes M. Mechanistic explanations how cell-mediated immune activation, inflammation and oxidative and nitrosative stress pathways and their sequels and concomitants play a role in the pathophysiology of unipolar depression. Neurosci Biobehav Rev. (2012) 36:764–85. doi: 10.1016/j.neubiorev.2011.12.005 22197082

[34] Xiao H Cao Y Lizano P Li M Sun H Zhou X. Interleukin-1β moderates the relationships between middle frontal-mACC/insular connectivity and depressive symptoms in bipolar II depression. Brain Behav Immun. (2024) 120:44–53. doi: 10.1016/j.bbi.2024.05.029 38777282

[35] Shen SY Liang LF Shi TL Shen ZQ Yin SY Zhang JR. Microglia-derived interleukin-6 triggers astrocyte apoptosis in the hippocampus and mediates depression-like behavior. Adv Sci (Weinh). (2025) 12:e2412556. doi: 10.1002/advs.202412556 39888279 PMC11923973

[36] Perry BI Upthegrove R Kappelmann N Jones PB Burgess S Khandaker GM. Associations of immunological proteins/traits with schizophrenia, major depression and bipolar disorder: A bi-directional two-sample mendelian randomization study. Brain Behav Immun. (2021) 97:176–85. doi: 10.1016/j.bbi.2021.07.009 PMC7612947 34280516

[37] Bahrini L Ouanes S Ghachem R. Inflammatory profile in depression and associated clinical and sociodemographic features in a Middle-Eastern North-African population. J Affect Disord. (2016) 198:122–6. doi: 10.1016/j.jad.2016.03.036 27015159

[38] Jingxuan M Yuzhen P Zhen L Juan W Hongjian W Yajia L. Monitoring of mental health in occupational populations: a study on the role and application of HDL-related inflammatory index. Front Public Health. (2025) 13:1563742. doi: 10.3389/fpubh.2025.1563742 40231184 PMC11994679

[39] Köhler CA Freitas TH Stubbs B Maes M Solmi M Veronese N. Peripheral alterations in cytokine and chemokine levels after antidepressant drug treatment for major depressive disorder: systematic review and meta-analysis. Mol Neurobiol. (2018) 55:4195–206. doi: 10.1007/s12035-017-0632-1 28612257

[40] Rethorst CD Toups MS Greer TL Nakonezny PA Carmody TJ Grannemann BD. Pro-inflammatory cytokines as predictors of antidepressant effects of exercise in major depressive disorder. Mol Psychiatry. (2013) 18:1119–24. doi: 10.1038/mp.2012.125 PMC3511631 22925832

