# Supplementary material for: Association between the Alpha-1-acid glycoprotein concentrations and depression in US adult women: a cross-sectional study from NHANES 2021–2023
Source: Front Psychiatry. 2025 Jun 3;16:1555321. doi: 10.3389/fpsyt.2025.1555321 (PMC12172548; doi:10.3389/fpsyt.2025.1555321)
Supplement: Supplementary file 1 [file Table1.doc]

**Table S1** Adjusted *P*-values for the Benjamini-Hochberg (BH) method.

|  | *P*_OR_-value | *P*_OR_-adjusted | *P*_β_-value | *P*_β_*-*adjusted |
| --- | --- | --- | --- | --- |
| Age |  |  |  |  |
| 20-34 years | 0.034 | 0.045 | 0.009 | 0.013 |
| 35-49 years | 0.128 | 0.163 | 0.303 | 0.346 |
| Race |  |  |  |  |
| Non-Hispanic White | 0.011 | 0.032 | 0.007 | 0.012 |
| Non-Hispanic Black | 0.454 | 0.502 | 0.282 | 0.323 |
| Mexican American | 0.183 | 0.237 | 0.181 | 0.239 |
| Other races | 0.566 | 0.611 | 0.589 | 0.636 |
| Educational levels |  |  |  |  |
| Less than High-school | 0.924 | 0.950 | 0.512 | 0.548 |
| High school | 0.174 | 0.232 | 0.176 | 0.224 |
| College or above | 0.023 | 0.037 | 0.017 | 0.031 |
| Poverty index ratio |  |  |  |  |
| PIR < 1 | 0.152 | 0.214 | 0.031 | 0.044 |
| 1 ≤ PIR < 3 | < 0.001 | < 0.001 | < 0.001 | < 0.001 |
| PIR > 3 | 0.457 | 0.502 | 0.178 | 0.239 |
| BMI |  |  |  |  |
| Underweight | 0.291 | 0.326 | 0.025 | 0.039 |
| Normal weight | 0.203 | 0.248 | 0.554 | 0.602 |
| Overweight | 0.484 | 0.532 | 0.463 | 0.511 |
| Obesity | 0.126 | 0.175 | 0.371 | 0.412 |
| Drinking status |  |  |  |  |
| Never | 0.950 | 0.950 | 0.836 | 0.865 |
| Former | 0.636 | 0.689 | 0.901 | 0.901 |
| Current | < 0.001 | <0.001 | < 0.001 | < 0.001 |
| Smoking status |  |  |  |  |
| Never | 0.031 | 0.043 | 0.004 | 0.010 |
| Former | 0.949 | 0.950 | 0.359 | 0.401 |
| Current | 0.067 | 0.114 | 0.312 | 0.376 |
| Physical levels |  |  |  |  |
| Vigorous | 0.012 | 0.025 | 0.033 | 0.047 |
| Middle | 0.170 | 0.214 | 0.146 | 0.194 |
| Other | 0.607 | 0.654 | 0.813 | 0.865 |
| Hypertension |  |  |  |  |
| Yes | 0.112 | 0.165 | 0.431 | 0.478 |
| No | 0.052 | 0.101 | 0.005 | 0.011 |
| Diabetes |  |  |  |  |
| Yes | 0.153 | 0.194 | 0.252 | 0.306 |
| No | 0.013 | 0.032 | 0.011 | 0.023 |
