# Supplementary material for: Association between the Alpha-1-acid glycoprotein concentrations and depression in US adult women: a cross-sectional study from NHANES 2021–2023
Source: Front Psychiatry. 2025 Jun 3;16:1555321. doi: 10.3389/fpsyt.2025.1555321 (PMC12172548; doi:10.3389/fpsyt.2025.1555321)
Supplement: Supplementary file 2 [file Table2.doc]

**Table S2** Results after sensitivity analyses.

|  | OR (95% CI) | *P*-value | *β* (95% CI) | *P*-value |
| --- | --- | --- | --- | --- |
| Crude model (Model Ⅰ) |  |  |  |  |
| Continuous | 2.73 (1.52, 4.71) | < 0.001 | 2.12 (1.08, 3.21) | < 0.001 |
| Q1 | *Ref* (1.00) |  | *Ref* (0.00) |  |
| Q2 | 1.34 (0.81, 2.23) | 0.232 | 0.63(-0.31, 1.65) | 0.287 |
| Q3 | 1.63 (0.85, 2.85) | 0.179 | 1.18 (-0.29, 2.11) | 0.364 |
| Q4 | 2.47 (1.36, 3.94) | < 0.001 | 1.97 (0.75, 3.15) | < 0.001 |
| *P* for trend | 0.003 |  | 0.002 |  |
| Partially adjusted model (Model Ⅱ) |  |  |  |  |
| Continuous | 2.29 (1.30, 3.86) | 0.001 | 1.79 (0.60 ,2.87) | < 0.001 |
| Q1 | *Ref* (1.00) |  | *Ref* (0.00) |  |
| Q2 | 1.26 (0.75, 2.34) | 0.247 | 0.59 (-0.38, 1.75) | 0.276 |
| Q3 | 1.51 (0.81, 2.52) | 0.128 | 1.01 (-0.31, 2.02) | 0.258 |
| Q4 | 1.92 (1.23, 3.48) | 0.002 | 1.58 (0.57, 2.49) | < 0.001 |
| *P* for trend | 0.004 |  | 0.003 |  |
| Fully adjusted model (Model Ⅲ) |  |  |  |  |
| Continuous | 1.94 (1.12, 3.43) | 0.006 | 1.36 (0.45, 2.42) | 0.008 |
| Q1 | *Ref* (1.00) |  | *Ref* (0.00) |  |
| Q2 | 1.18 (0.72, 2.02) | 0.412 | 0.49 (-0.52,1.47) | 0.310 |
| Q3 | 1.43 (0.76, 2.36) | 0.354 | 0.91 (-0.46, 1.85) | 0.248 |
| Q4 | 1.83 (1.18, 2.73) | 0.003 | 1.41 (0.27, 2.23) | 0.005 |
| *P* for trend | 0.008 |  | 0.009 |  |
